# Ethical acceptability of research on human-animal chimeric embryos: summary of opinions by the Japanese Expert Panel on Bioethics

**DOI:** 10.1186/s40504-015-0033-z

**Published:** 2015-12-22

**Authors:** Hiroshi Mizuno, Hidenori Akutsu, Kazuto Kato

**Affiliations:** Agrogenomics Research Center, National Institute of Agrobiological Sciences, Tsukuba, 305-8602 Japan; Department of Reproductive Medicine, National Research Institute for Child Health and Development, 2-10-1 Okura, Setagaya-ku, Tokyo 157-8535 Japan; Department of Biomedical Ethics and Public Policy, Graduate School of Medicine, Osaka University, Suita, 565-0871 Japan; Institute for Integrated Cell-Material Sciences, Kyoto University, Kyoto, 606-8501 Japan

**Keywords:** ELSI, Human organ, Pluripotency, Embryonic stem cell (ESC), Induced pluripotent stem cell (iPSC), Human dignity, Chimerization

## Abstract

Human-animal chimeric embryos are embryos obtained by introducing human cells into a non-human animal embryo. It is envisaged that the application of human-animal chimeric embryos may make possible many useful research projects including producing three-dimensional human organs in animals and verification of the pluripotency of human ES cells or iPS cells in vivo. The use of human-animal chimeric embryos, however, raises several ethical and moral concerns. The most fundamental one is that human-animal chimeric embryos possess the potential to develop into organisms containing human-derived tissue, which may lead to infringing upon the identity of the human species, and thus impairing human dignity. The Japanese Expert Panel on Bioethics in the Cabinet Office carefully considered the scientific significance and ethical acceptability of the issue and released its “Opinions regarding the handling of research using human-animal chimeric embryos”. The Panel proposed a framework of case-by-case review, and suggested that the following points must be carefully reviewed from the perspective of ethical acceptability: (a) Types of animal embryos and types of animals receiving embryo transfers, particularly in dealing with non-human primates; (b) Types of human cells and organs intended for production, particularly in dealing with human nerve or germ cells; and (c) Extent of the period required for post-transfer studies. The scientific knowledge that can be gained from transfer into an animal uterus and from the production of an individual must be clarified to avoid unnecessary generation of chimeric animals. The time is ripe for the scientific community and governments to start discussing the ethical issues for establishing a global consensus.

## Introduction

Human-animal chimeric embryos are created by introducing human cells into a non-human animal embryo. One possible application of this research is the use of animals to produce three-dimensional human organs in animals, which would produce animal models of human diseases and supply human organs available for transplantation (Rashid et al. 2014). Recent scientific progress may make such research a reality. “Animal-Animal” chimeric embryos have been shown to develop into a target organ by using blastocyst complementation (Kobayashi et al. 2010; Matsunari et al. 2013). Another application is the verification of pluripotency of human putative pluripotent stem cells induced by molecular reprogramming. Although two phases of pluripotency can be defined: naïve and primed (Nichols and Smith 2009), the regulatory principles of pluripotency have not fully been elucidated (Hackett and Surani 2014). These advances in resetting human pluripotent stem cells and demonstrating the subsequent formation of interspecies chimeric embryos suggest that research in this field may expand more rapidly than we currently imagine.

The use of human-animal chimeric embryos, however, raises several ethical and moral concerns. The most fundamental one is that such embryos possess the potential to develop into organisms containing human-derived tissue, which may lead to a blurring of the identity of the human species. Human-animal chimeras, such as those generated when transplanting human tumor cells into mice or creating a model mouse of the human immune system (Hyun et al. 2007), have been used as important tools for biomedical research. However, the use of human-animal chimeric embryos raises a serious ethical concern because it is impossible to predict fully how human pluripotent stem cells will develop in animal bodies. Such organisms could blur the boundary between human and non-human organisms, if a significant number of human brain (nerve) or germ cells were to function appropriately in animal bodies. Other concerns include the use of non-human primates and procedures to ensure the welfare of animals used in such research. A careful debate on the scientific significance and ethical acceptability of studies that involve human-animal chimeric embryos is thus required.

Since 2012, the Japanese Expert Panel on Bioethics has discussed the ethical issues regarding research that involves human-animal chimeric embryos, held hearings with outside experts, and carried out surveys of research trends and regulations in other countries. The panel is one of the expert panels under the Council for Science, Technology and Innovation in the Cabinet Office; it investigates and deliberates on basic policies that relate to ethical issues that arise from the rapid development of the life sciences; it consists of medical doctors, journalists specialized in medical fields, as well as academic investigators in stem cell research, law, and bioethics. The panel released its “Opinions regarding the handling of research that involves human-animal chimeric embryos” in 2013 to state its recommendations for revising the governmental guidelines. In light of the opinion, detailed discussions are underway within the Ministry of Education, Culture, Sports, Science and Technology (MEXT) for categorizing possible research projects involving human-animal chimeric embryos.

In this paper, we describe scientific significance and ethical acceptability on research that involve human-animal chimeric embryos—especially summary of discussion held by the Japanese Expert Panel on Bioethics with regard to conditions for approving transfer an human-animal chimeric embryo into an animal uterus. It is hoped that the debate in Japan will be useful for the creation of research frameworks in other countries.

### Guidelines and current status of relevant research

More than a decade ago, Japan established the framework on research that involves human-animal chimeric embryos. In 2000, the Act on Regulation of Human Cloning Techniques came into effect. In 2001, the Guidelines for Handling of a Specified Embryo were formulated on the basis of this law, which include the following stipulations regarding human-animal chimeric embryos:

1. They may only be produced for the purpose of basic research concerning “human cell-derived organs transplantable to a human being”;
2. They may only be cultured until the appearance of the primitive streak (maximum 14 days);
3. For the time being, their transfer into a human or animal uterus is prohibited.

The prohibition on the transfer into a uterus is intended to prevent the production of individuals in which the boundary between animal and human is blurred, and is based on the Resolution of the Diet “The transfer of a human-animal chimeric embryo into a human or animal uterus should be prohibited as long as there is the risk that it might affect the preservation of human dignity in a manner equivalent to that of a human clone or hybrid individual” (A Supplementary Resolution of the Diet of the Act on Regulation of Human Cloning Techniques in 2000). These regulations are fundamentally based on the objective of the Act: “to preserve human dignity, ensure the safety of each human life and body, and maintain public order”.

Under the present guidelines in Japan, basic research is being conducted on producing organs from human iPS cells. Nakauchi and colleagues have evaluated the chimera-forming ability of human pluripotent stem cells in vitro (Masaki et al. 2015). Their culturing of human-animal chimeric embryos is permitted in vitro until the appearance of the primitive streak, which precedes the start of tissue differentiation. If the aim is to produce three-dimensional human organs and evaluate their function, however, it will be necessary to transfer chimeric embryos into an animal uterus, culture them after the appearance of the primitive streak (more than 14 days in total), and allow the organ to develop to a certain size in vivo. These additional steps might raise ethical concerns of generating human-animal chimeras.

### Discussion held by the Japanese Expert Panel on Bioethics

Taking into account the ethical concerns and recent scientific progress described above, the Panel considered the acceptable conditions for research that involves transferring human-animal chimeric embryos into animal uterus, and proposed that:

1. Measures be used to prevent the unnecessary generation of individual animals;
2. If the generation of individuals is approved, there be no risk that the individuals might develop into organisms that violate “human dignity” in the sense of infringing upon the identity of the human species;
3. Studies must possess both scientific significance and social acceptability”.

To prevent the generation of individuals that might infringe upon the identity of the human species, it will therefore be necessary to explain scientifically that the individuals will neither be human nor human-like organisms. It is thus important to ensure accuracy of techniques for differentiating human stem cells into a specific tissue or organ in animals. Regarding the precision of techniques to ensure that a human-animal chimeric embryo develops only into the target organ, much progress has been made since the guidelines were formulated in 2001. Techniques to control differentiation, which involve animals genetically lacking in specific organs, have been used to produce (1) rat pancreas in the bodies of pancreatogenesis-disabled mice and vice versa (Kobayashi et al. 2010), as well as (2) pancreas derived from healthy pigs in pancreatogenesis-disabled pigs as a model of large livestock animals (Matsunari et al. 2013). In addition, cutting-edge genetic manipulation techniques are used to induce differentiation selectively toward the endodermal lineage (Kobayashi et al. 2015), and might be used to introduce suicide genes under neuron– or germ cell–specific promoters to induce apoptosis of unwanted cells after differentiation (Rashid et al. 2014). However, it is necessary to consider how to respond to unintended events in the course of the research project in advance.

Irrespective of the accuracy of differentiation control techniques, however, limitations should be imposed from the viewpoint of ethical acceptability. The Panel proposed that the following points must be carefully considered to approve the transfer of a human-animal chimeric embryo into an animal uterus:

1. Types of animal embryos and types of animal recipients.

Studies of a non-human primate will require particular caution, both from the perspective of blurring the boundary between animals and humans and from the viewpoint of animal welfare;

(2) Types of human cells used in chimeras or types of human cells and organs intended for production.

In particular, producing a brain derived from human cells in an animal body may have an effect on animal behavior, and should be regulated even at stages before individuals are generated;

(3) Extent of the period required for research after transfer into an animal uterus.

The period will vary depending on matters including the study objective, type of animal used, and organ to be produced. It will therefore be necessary to clarify what scientific knowledge can be acquired in accordance with each individual study. When generating an individual, the necessity of generating an individual should be considered upon clarifying the scientific knowledge that can be obtained as well as the conditions for approving transfer into an animal uterus.

When debating whether or not to permit the transfer of a human-animal chimeric embryo to an animal uterus, the panel was aware that such transfer would comprise the first step in numerous future research projects. It still remains unclear whether or not human ES or iPS cells have the potential to form interspecies chimeras, and it is unknown whether or not a human-animal chimeric embryo is even capable of undergoing initial development in vivo. Injection of human pluripotent stem cells into mouse blastocysts and in vitro culture did not result in human-mouse chimera formation (Masaki et al. 2015), whereas region-selective human pluripotent stem cells undergo interspecies chimerization ex vivo by using epiblasts isolated from mice (Wu et al. 2015). Given that highly valuable research projects will likely be envisaged with advances in basic research, it will also be necessary to engage a wider range of stakeholders, including patients and citizens, and to discuss what kind of research is acceptable in society.

### Establishing a regulatory framework in Japan

To situate the opinions of the Expert Panel within the regulatory framework in Japan, the current guideline (Guidelines for Handling of Specified Embryos 2001), but not the act (Act on Regulation of Human Cloning Techniques 2000), will need to be revised in terms of the following points: (1) repealing the prohibition on transferring embryos into an animal uterus, and (2) establishing a framework for case-by-case review by stipulating unacceptable research purposes. The panel also pointed out that the use of human-animal chimeric embryos may enable many useful research projects, and that the purposes of research (limited under the current guidelines to basic research concerning the production of human organs) should therefore be expanded. Because it is impossible to predict future research developments with any certainty, the purposes of producing human-animal chimeric embryos should not be limited too strictly by law or other regulations. Therefore, the panel proposed a system whereby the regulatory framework indicates clearly unacceptable research purposes (for example, generation of functional human brain in non-human primates, and unnecessary generation of individual animals), and research projects are approved on a case-by-case basis. The review process for basic research is currently under the jurisdiction of MEXT. In addition, the Ministry of Health, Labour, and Welfare will also be involved in the case of clinical studies in the future. Ensuring the efficacy and safety needed to use organs/tissues derived from human-animal chimeric embryos in clinical studies will require compliance with the “Act on the Safety of Regenerative Medicine” and “The Pharmaceuticals, Medical Devices, and Other Therapeutic Products Act” (Konomi et al. 2015). These laws and guidelines will constitute the legal framework for research involving human-animal chimeric embryos, from basic research to studies on the use of such embryos for medical purposes.

### Current landscape of regulations in the UK, USA, and by the ISSCR

Research involving human-animal chimeric embryos has also been discussed in other countries. In the United States, the National Institutes of Health released a set of guidelines in 2009 (NIH 2009) based on the recommendations of the National Research Council and the Institute of Medicine of the U.S. National Academies (2005). In the United Kingdom, a report titled “Animals Containing Human Material (ACHM)” has been released (The Academy of Medical Sciences 2011). The International Society for Stem Cell Research (ISSCR) has also considered this issue and released a draft of its guidelines (ISSCR 2015).

These guidelines and reports provide important views that have not been addressed in the Japanese opinion. With regard to the cells, tissues, or organs that may be produced, “the degree” to which human nerve cells become functionally integrated in an animal’s central nervous system (ISSCR 2015), and the “breeding” of animal chimeras that form human gametes (NIH 2009; ISSCR 2015), are important factors for evaluating ethical concerns. This implies that a complete prohibition of nerve- or germ-cell generation in animals might not be necessary. The ACHM provides categories of human-animal chimeras whose production should be restricted and points out the concern of altering “appearances” or behaviors that distinguish humans from other species (e.g., humanized skin, limb, or facial structure; Rashid et al. 2014). With regard to the animal species used, transfer of human ES or iPS cells into non-human primate blastocysts is prohibited in studies supported by the National Institutes of Health (NIH 2009). With regard to transfer into an animal uterus, an embryo equivalent to a human-animal chimeric embryo may be transferred into an animal uterus and allowed to develop until halfway through the gestation period, with individual screening of research projects as a prerequisite in the UK (Mizuho Information and Research Institute, Inc. 2013). Japan has emphasized the importance of avoiding unnecessary generation of individual animals in the present opinions. Consequently, the scientific knowledge that can be gained in accordance with each step of the experiment in vivo needs to be clarified to keep the culturing period as short as possible.

Human-animal chimeric embryos are presently dealt with inconsistently in different countries, with no clear consensus on their prohibition or acceptance. The time is ripe for the scientific community and governments to start discussing the ethical issue.

## Conclusion

1. Looking at the current progress of research on human stem cells and human-animal chimeric embryos, it may be possible to acquire new scientific knowledge on the following matters with transfer human-animal chimeric embryos into an animal uterus.
  - Confirmation of whether or not it is actually feasible to create organs from human stem cells, whether or not they can be shaped, and whether or not they possess the functions;
  - Creation of novel animal models having human tissues/organs for elucidating the pathologies of human disorders, including those that are currently intractable;
  - Verification of the pluripotency of human putative pluripotent stem cells.
2. Given that highly valuable applications will likely be envisaged with advances in research, regulations on the research purpose should be capable of responding flexibly to the advances in research. In addition to the “positive list” format of the current regulation in Japan, which lists acceptable research purposes, it is appropriate to consider the additional use of a “negative list” format that lists unacceptable research purposes, or a shift to the negative list format completely. It is thus necessary to set up and operate a framework for conducting a review for each study protocol. It is also necessary to discuss what kind of research is acceptable in society.
3. It is necessary to formulate conditions to ensure that there is no risk of violating human dignity in the sense of infringing upon the identity of the human species. Irrespective of the accuracy of differentiation control techniques, the following points must be carefully considered from the viewpoint of ethical acceptability: types of animal embryos and types of animals receiving embryo transfers; types of human cells used in chimeras or types of human cells and organs intended for production; extent of the period required for research after transfer into an animal uterus.

We consider the observations and recommendations by the Japanese Expert Panel on Bioethics to be useful for the formulation of regulatory frameworks in other scientific communities and governments.
